# A monoclinic polymorph of theophylline

**DOI:** 10.1107/S1600536811047532

**Published:** 2011-11-19

**Authors:** Shuo Zhang, Andreas Fischer

**Affiliations:** a Division of Transport Phenomena, School of Chemical Science and Engineering, Royal Institute of Technology (KTH), 100 44 Stockholm, Sweden; b Inorganic Chemistry, School of Chemical Science and Engineering, Royal Institute of Technology (KTH), 100 44 Stockholm, Sweden

## Abstract

A monoclinic polymorph of theophylline, C_7_H_8_N_4_O_2_, has been obtained from a chloro­form/methanol mixture by evaporation under ambient conditions. The new polymorph crystallizes with two mol­ecules in the asymmetric unit. The structure features inter­molecular N—H⋯O hydrogen bonds, resulting in the formation of dimers between two crystallographically different mol­ecules; each mol­ecule acts as both donor and acceptor.

## Related literature

For the ortho­rhom­bic polymorph of anhydrous theophylline, see: Ebisuzaki *et al.* (1997 ▶).
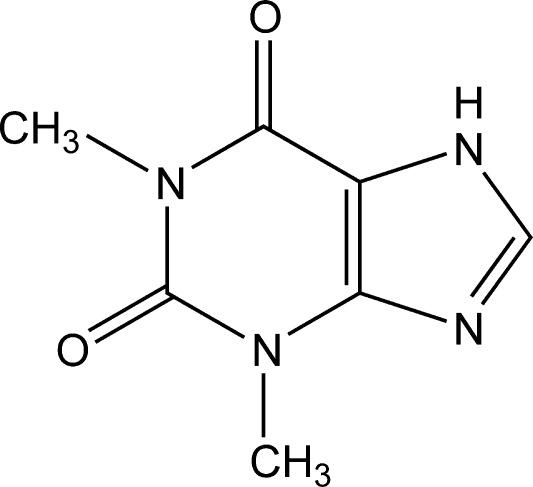


## Experimental

### 

#### Crystal data

C_7_H_8_N_4_O_2_
*M* *_r_* = 180.17
Monoclinic, 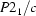

*a* = 7.8935 (6) Å
*b* = 12.9087 (7) Å
*c* = 15.9055 (8) Å
β = 104.214 (5)°
*V* = 1571.07 (17) Å^3^
*Z* = 8
Mo *K*α radiation
μ = 0.12 mm^−1^
*T* = 299 K
0.35 × 0.29 × 0.04 mm

#### Data collection

Bruker–Nonius KappaCCD diffractometer
Absorption correction: multi-scan (*SADABS*; Sheldrick, 2003 ▶) *T*_min_ = 0.863, *T*_max_ = 0.995
19027 measured reflections
3074 independent reflections
1972 reflections with *I* > 2σ(*I*)
*R*_int_ = 0.058

#### Refinement

*R*[*F* ^2^ > 2σ(*F* ^2^)] = 0.048
*wR*(*F* ^2^) = 0.129
*S* = 1.05
3074 reflections
239 parameters
H-atom parameters constrained
Δρ_max_ = 0.22 e Å^−3^
Δρ_min_ = −0.24 e Å^−3^

### 

Data collection: *COLLECT* (Nonius, 1998 ▶); cell refinement: *DIRAX* (Duisenberg, 1992 ▶); data reduction: *EVALCCD* (Duisenberg *et al.*, 2003 ▶); program(s) used to solve structure: *SHELXS97* (Sheldrick, 2008 ▶); program(s) used to refine structure: *SHELXL97* (Sheldrick, 2008 ▶); molecular graphics: *DIAMOND* (Brandenburg, 2007 ▶); software used to prepare material for publication: *publCIF* (Westrip, 2010 ▶).

## Supplementary Material

Crystal structure: contains datablock(s) global, I. DOI: 10.1107/S1600536811047532/wn2455sup1.cif

Structure factors: contains datablock(s) I. DOI: 10.1107/S1600536811047532/wn2455Isup2.hkl

Supplementary material file. DOI: 10.1107/S1600536811047532/wn2455Isup3.cml

Additional supplementary materials:  crystallographic information; 3D view; checkCIF report

## Figures and Tables

**Table 1:** Hydrogen-bond geometry (Å, °)

*D*—H⋯*A*	*D*—H	H⋯*A*	*D*⋯*A*	*D*—H⋯*A*
N4—H4⋯O3^i^	0.86	1.92	2.753 (2)	163
N8—H8⋯O1^i^	0.86	1.94	2.782 (2)	165

Symmetry code: (i) 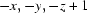.

**Fig. 1.**
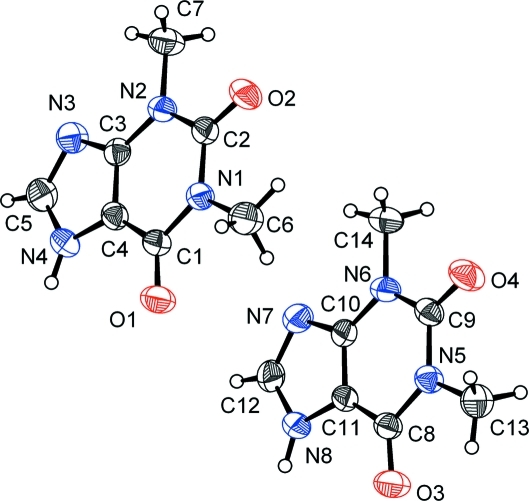
The asymmetric unit of the title compound. Displacement ellipsoids are drawn at the 50% probability level. Hydrogen atoms are shown as spheres of arbitrary radius.

**Fig. 2.**
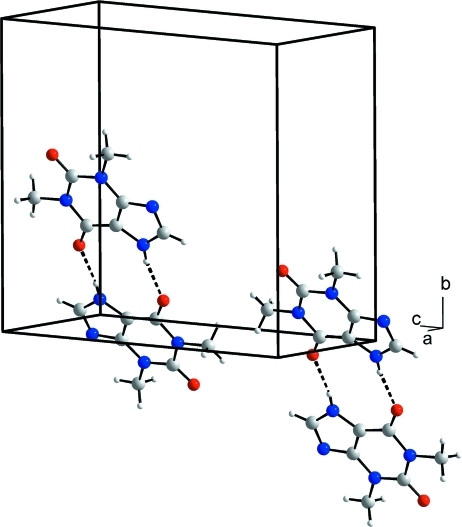
The hydrogen bonding pattern in the title compound, yielding dimers. Hydrogen bonds are indicated as dashed lines.

## supplementary crystallographic information

### Comment

Theophylline,1,3-dimethyl-7*H*-purine-2,6-dione, is an FDA-approved compound for the treatment of respiratory diseases such as asthma, as are other related compounds, such as caffeine and theobromine.

Thus far, only one crystal structure of anhydrous theophylline has been reported (Ebisuzaki *et al.*, 1997). This orthorhombic structure has one molecule per asymmetric unit and is characterized by N—H···N hydrogen bonds, yielding infinite chains.

The polymorph of theophylline in this work was found during a solubility study using a 4:1 mixture ofchloroform and methanol as solvent. This monoclinic polymorph has been found to be thermodynamically stable at room temperature. Solubility data and a discussion of thermodynamic stability relationships will be presented elsewhere. (Zhang & Rasmuson, manuscript in preparation).

The title compound features two molecules in the asymmetric unit (Fig. 1). They are almost coplanar with a dihedral angle of 5.31 (3)°. Each molecule acts as N—H···O bond donor and acceptor, yielding dimers of two crystallographically different molecules (Fig. 2).

The major difference between the structures of the two polymorphs is their hydrogen bonding pattern.

### Experimental

Commercial theophylline was dissolved in a mixture of chloroform and methanol (ratio 4:1 *v*/*v*). Evaporation at ambient temperature and pressure over a period of five weeks yielded the title compound. Powder X-ray diffraction confirmed that the bulk material was identical with the single-crystal from which the crystal structure was obtained.

### Refinement

H atoms were placed at calculated positions and refined as riding, with N—H = 0.86 Å, C(methyl)—H = 0.96 Å and Csp^2^—H = 0.93 Å. *U*_iso_(H) = x*U*_eq_(C,N), where x = 1.5 for methyl H and 1.2 for all other H atoms.

### Crystal data

**Table:**

C_7_H_8_N_4_O_2_	*F*(000) = 752
*M**_r_* = 180.17	*D*_x_ = 1.523 Mg m^−^^3^
Monoclinic, *P*2_1_/*c*	Mo *K*α radiation, λ = 0.71073 Å
Hall symbol: -P 2ybc	Cell parameters from 82 reflections
*a* = 7.8935 (6) Å	θ = 4.2–20.8°
*b* = 12.9087 (7) Å	µ = 0.12 mm^−^^1^
*c* = 15.9055 (8) Å	*T* = 299 K
β = 104.214 (5)°	Plate, colourless
*V* = 1571.07 (17) Å^3^	0.35 × 0.29 × 0.04 mm
*Z* = 8	

### Data collection

**Table:**

Bruker–Nonius KappaCCD diffractometer	1972 reflections with *I* > 2σ(*I*)
Radiation source: fine-focus sealed tube	*R*_int_ = 0.058
ω scans	θ_max_ = 26.0°, θ_min_ = 4.5°
Absorption correction: multi-scan (*SADABS*; Sheldrick, 2003)	*h* = −9→8
*T*_min_ = 0.863, *T*_max_ = 0.995	*k* = −15→15
19027 measured reflections	*l* = −19→19
3074 independent reflections	

### Refinement

**Table:**

Refinement on *F*^2^	Primary atom site location: structure-invariant direct methods
Least-squares matrix: full	Secondary atom site location: difference Fourier map
*R*[*F*^2^ > 2σ(*F*^2^)] = 0.048	H-atom parameters constrained
*wR*(*F*^2^) = 0.129	*w* = 1/[σ^2^(*F*_o_^2^) + (0.0593*P*)^2^ + 0.4941*P*] where *P* = (*F*_o_^2^ + 2*F*_c_^2^)/3
*S* = 1.05	(Δ/σ)_max_ < 0.001
3074 reflections	Δρ_max_ = 0.22 e Å^−^^3^
239 parameters	Δρ_min_ = −0.24 e Å^−^^3^
0 restraints	

### Special details

**Table:**

Geometry. All e.s.d.'s (except the e.s.d. in the dihedral angle between two l.s. planes) are estimated using the full covariance matrix. The cell e.s.d.'s are taken into account individually in the estimation of e.s.d.'s in distances, angles and torsion angles; correlations between e.s.d.'s in cell parameters are only used when they are defined by crystal symmetry. An approximate (isotropic) treatment of cell e.s.d.'s is used for estimating e.s.d.'s involving l.s. planes.
Refinement. Refinement of *F*^2^ against ALL reflections. The weighted *R*-factor *wR* and goodness of fit *S* are based on *F*^2^, conventional *R*-factors *R* are based on *F*, with *F* set to zero for negative *F*^2^. The threshold expression of *F*^2^ > σ(*F*^2^) is used only for calculating *R*-factors(gt) *etc*. and is not relevant to the choice of reflections for refinement. *R*-factors based on *F*^2^ are statistically about twice as large as those based on *F*, and *R*- factors based on ALL data will be even larger.

### Fractional atomic coordinates and isotropic or equivalent isotropic displacement parameters (Å^2^)

**Table:**

	*x*	*y*	*z*	*U*_iso_*/*U*_eq_	
C1	0.3056 (3)	0.02115 (17)	0.09461 (14)	0.0352 (5)	
C2	0.4877 (3)	0.17786 (17)	0.09060 (14)	0.0342 (5)	
C3	0.3328 (3)	0.09963 (17)	−0.04057 (13)	0.0316 (5)	
C4	0.2672 (3)	0.02566 (17)	0.00340 (14)	0.0336 (5)	
C5	0.1763 (3)	0.0045 (2)	−0.13534 (15)	0.0435 (6)	
C6	0.4624 (4)	0.1056 (2)	0.22819 (15)	0.0554 (7)	
C7	0.5185 (3)	0.2516 (2)	−0.04623 (16)	0.0489 (6)	
C8	0.0635 (3)	0.28631 (17)	1.02459 (14)	0.0337 (5)	
C9	0.2209 (3)	0.45280 (17)	1.02194 (14)	0.0351 (5)	
C10	0.0752 (3)	0.37253 (17)	0.89022 (13)	0.0324 (5)	
C11	0.0220 (3)	0.29279 (16)	0.93328 (13)	0.0314 (5)	
C12	−0.0732 (3)	0.27496 (19)	0.79473 (14)	0.0425 (6)	
C13	0.2196 (3)	0.3724 (2)	1.15919 (14)	0.0475 (6)	
C14	0.2368 (3)	0.53625 (19)	0.88634 (16)	0.0471 (6)	
N1	0.4160 (2)	0.10126 (14)	0.13328 (11)	0.0361 (5)	
N2	0.4434 (2)	0.17564 (14)	0.00173 (11)	0.0346 (4)	
N3	0.2779 (3)	0.08774 (15)	−0.12768 (12)	0.0418 (5)	
N4	0.1653 (2)	−0.03563 (15)	−0.05995 (12)	0.0391 (5)	
N5	0.1646 (2)	0.36962 (14)	1.06438 (11)	0.0345 (4)	
N6	0.1742 (2)	0.45278 (14)	0.93298 (12)	0.0366 (5)	
N7	0.0172 (3)	0.36232 (15)	0.80311 (12)	0.0433 (5)	
N8	−0.0753 (2)	0.23019 (15)	0.86970 (11)	0.0366 (5)	
O1	0.2529 (2)	−0.04282 (13)	0.13937 (10)	0.0497 (5)	
O2	0.5851 (2)	0.24386 (13)	0.13070 (10)	0.0484 (4)	
O3	0.0208 (2)	0.21730 (13)	1.06858 (10)	0.0496 (5)	
O4	0.3076 (2)	0.52232 (13)	1.06289 (11)	0.0514 (5)	
H5	0.1181	−0.0232	−0.1886	0.052*	
H6A	0.5544	0.1552	0.2475	0.083*	
H6B	0.5013	0.0386	0.2512	0.083*	
H6C	0.3619	0.1259	0.2481	0.083*	
H7A	0.4913	0.3201	−0.0300	0.073*	
H7B	0.4707	0.2419	−0.1073	0.073*	
H7C	0.6430	0.2429	−0.0330	0.073*	
H12	−0.1301	0.2474	0.7412	0.051*	
H13A	0.3448	0.3691	1.1774	0.071*	
H13B	0.1704	0.3143	1.1825	0.071*	
H13C	0.1797	0.4355	1.1798	0.071*	
H14A	0.2042	0.6019	0.9061	0.071*	
H14B	0.1856	0.5293	0.8253	0.071*	
H14C	0.3617	0.5324	0.8970	0.071*	
H4	0.1062	−0.0893	−0.0523	0.047*	
H8	−0.1272	0.1735	0.8769	0.044*	

### Atomic displacement parameters (Å^2^)

**Table:**

	*U*^11^	*U*^22^	*U*^33^	*U*^12^	*U*^13^	*U*^23^
C1	0.0348 (11)	0.0294 (13)	0.0408 (12)	−0.0032 (10)	0.0079 (10)	0.0011 (10)
C2	0.0323 (12)	0.0308 (12)	0.0378 (12)	−0.0009 (10)	0.0052 (9)	−0.0005 (10)
C3	0.0316 (11)	0.0286 (12)	0.0336 (11)	0.0031 (9)	0.0061 (9)	−0.0018 (9)
C4	0.0315 (11)	0.0301 (13)	0.0378 (12)	−0.0008 (9)	0.0059 (9)	−0.0033 (10)
C5	0.0490 (14)	0.0433 (15)	0.0358 (13)	−0.0034 (12)	0.0054 (10)	−0.0076 (11)
C6	0.0696 (18)	0.0563 (18)	0.0351 (13)	−0.0173 (14)	0.0034 (12)	0.0011 (12)
C7	0.0568 (15)	0.0423 (15)	0.0501 (14)	−0.0130 (12)	0.0182 (12)	0.0037 (12)
C8	0.0329 (11)	0.0294 (12)	0.0391 (12)	−0.0022 (9)	0.0093 (9)	−0.0004 (10)
C9	0.0343 (11)	0.0301 (13)	0.0412 (13)	−0.0035 (10)	0.0098 (10)	−0.0047 (10)
C10	0.0298 (11)	0.0312 (12)	0.0347 (11)	0.0018 (9)	0.0049 (9)	0.0014 (10)
C11	0.0309 (11)	0.0268 (12)	0.0348 (12)	−0.0019 (9)	0.0046 (9)	−0.0016 (9)
C12	0.0443 (13)	0.0419 (15)	0.0354 (13)	−0.0028 (11)	−0.0018 (10)	−0.0015 (11)
C13	0.0569 (15)	0.0482 (16)	0.0349 (12)	−0.0065 (13)	0.0063 (11)	−0.0055 (11)
C14	0.0518 (15)	0.0378 (15)	0.0526 (15)	−0.0097 (11)	0.0147 (12)	0.0067 (11)
N1	0.0400 (10)	0.0336 (11)	0.0318 (10)	−0.0063 (8)	0.0030 (8)	−0.0002 (8)
N2	0.0377 (10)	0.0297 (10)	0.0356 (10)	−0.0049 (8)	0.0074 (8)	0.0007 (8)
N3	0.0493 (11)	0.0394 (12)	0.0359 (10)	−0.0039 (9)	0.0088 (9)	−0.0042 (9)
N4	0.0390 (10)	0.0334 (11)	0.0428 (11)	−0.0084 (8)	0.0060 (9)	−0.0048 (9)
N5	0.0386 (10)	0.0321 (11)	0.0321 (9)	−0.0047 (8)	0.0073 (8)	−0.0038 (8)
N6	0.0413 (11)	0.0280 (10)	0.0404 (11)	−0.0055 (8)	0.0096 (8)	0.0020 (8)
N7	0.0495 (12)	0.0381 (12)	0.0377 (11)	−0.0044 (10)	0.0021 (9)	0.0034 (9)
N8	0.0385 (10)	0.0301 (11)	0.0384 (10)	−0.0073 (8)	0.0040 (8)	−0.0013 (8)
O1	0.0596 (11)	0.0450 (11)	0.0429 (10)	−0.0183 (8)	0.0099 (8)	0.0047 (8)
O2	0.0516 (10)	0.0408 (10)	0.0483 (10)	−0.0171 (8)	0.0034 (8)	−0.0057 (8)
O3	0.0661 (11)	0.0410 (10)	0.0410 (9)	−0.0177 (9)	0.0116 (8)	0.0023 (8)
O4	0.0599 (11)	0.0405 (10)	0.0518 (10)	−0.0173 (9)	0.0098 (8)	−0.0104 (8)

### Geometric parameters (Å, °)

**Table:**

C1—O1	1.228 (3)	C10—N6	1.373 (3)
C1—N1	1.394 (3)	C11—N8	1.373 (3)
C1—C4	1.409 (3)	C12—N7	1.324 (3)
C2—O2	1.218 (3)	C12—N8	1.329 (3)
C2—N2	1.370 (3)	C13—N5	1.464 (3)
C2—N1	1.396 (3)	C14—N6	1.461 (3)
C3—N3	1.355 (3)	C5—H5	0.9300
C3—C4	1.359 (3)	C6—H6A	0.9600
C3—N2	1.374 (3)	C6—H6B	0.9600
C4—N4	1.375 (3)	C6—H6C	0.9600
C5—N4	1.328 (3)	C7—H7A	0.9600
C5—N3	1.329 (3)	C7—H7B	0.9600
C6—N1	1.465 (3)	C7—H7C	0.9600
C7—N2	1.455 (3)	C12—H12	0.9300
C8—O3	1.230 (3)	C13—H13A	0.9600
C8—N5	1.395 (3)	C13—H13B	0.9600
C8—C11	1.411 (3)	C13—H13C	0.9600
C9—O4	1.216 (3)	C14—H14A	0.9600
C9—N6	1.372 (3)	C14—H14B	0.9600
C9—N5	1.398 (3)	C14—H14C	0.9600
C10—N7	1.355 (3)	N4—H4	0.8600
C10—C11	1.359 (3)	N8—H8	0.8600
			
O1—C1—N1	120.46 (19)	C9—N6—C10	119.16 (18)
O1—C1—C4	127.4 (2)	C9—N6—C14	118.99 (19)
N1—C1—C4	112.14 (19)	C10—N6—C14	121.81 (19)
O2—C2—N2	121.5 (2)	C12—N7—C10	102.95 (19)
O2—C2—N1	121.4 (2)	C12—N8—C11	106.07 (19)
N2—C2—N1	117.11 (19)	N4—C5—H5	123.1
N3—C3—C4	112.32 (19)	N3—C5—H5	123.1
N3—C3—N2	125.9 (2)	N1—C6—H6A	109.5
C4—C3—N2	121.75 (19)	N1—C6—H6B	109.5
C3—C4—N4	104.81 (18)	H6A—C6—H6B	109.5
C3—C4—C1	123.1 (2)	N1—C6—H6C	109.5
N4—C4—C1	132.1 (2)	H6A—C6—H6C	109.5
N4—C5—N3	113.8 (2)	H6B—C6—H6C	109.5
O3—C8—N5	120.43 (19)	N2—C7—H7A	109.5
O3—C8—C11	127.0 (2)	N2—C7—H7B	109.5
N5—C8—C11	112.55 (19)	H7A—C7—H7B	109.5
O4—C9—N6	121.7 (2)	N2—C7—H7C	109.5
O4—C9—N5	120.8 (2)	H7A—C7—H7C	109.5
N6—C9—N5	117.47 (19)	H7B—C7—H7C	109.5
N7—C10—C11	111.92 (19)	N7—C12—H12	123.0
N7—C10—N6	126.0 (2)	N8—C12—H12	123.0
C11—C10—N6	122.04 (19)	N5—C13—H13A	109.5
C10—C11—N8	105.13 (18)	N5—C13—H13B	109.5
C10—C11—C8	122.8 (2)	H13A—C13—H13B	109.5
N8—C11—C8	132.1 (2)	N5—C13—H13C	109.5
N7—C12—N8	113.9 (2)	H13A—C13—H13C	109.5
C1—N1—C2	126.52 (17)	H13B—C13—H13C	109.5
C1—N1—C6	117.04 (18)	N6—C14—H14A	109.5
C2—N1—C6	116.44 (18)	N6—C14—H14B	109.5
C2—N2—C3	119.36 (18)	H14A—C14—H14B	109.5
C2—N2—C7	119.55 (19)	N6—C14—H14C	109.5
C3—N2—C7	121.06 (18)	H14A—C14—H14C	109.5
C5—N3—C3	102.70 (19)	H14B—C14—H14C	109.5
C5—N4—C4	106.34 (19)	C5—N4—H4	126.8
C8—N5—C9	125.98 (18)	C4—N4—H4	126.8
C8—N5—C13	118.55 (18)	C12—N8—H8	127.0
C9—N5—C13	115.47 (18)	C11—N8—H8	127.0

### Hydrogen-bond geometry (Å, °)

**Table:**

*D*—H···*A*	*D*—H	H···*A*	*D*···*A*	*D*—H···*A*
N4—H4···O3^i^	0.86	1.92	2.753 (2)	163
N8—H8···O1^i^	0.86	1.94	2.782 (2)	165

Symmetry codes: (i) −*x*, −*y*, −*z*+1.
